# UHPLC-Q-TOF MS-Based Metabolic Analysis for the Therapeutic Efficacy of “Xuebijing Injection” against Sepsis-Induced Acute Lung Injury

**DOI:** 10.1155/2018/8514619

**Published:** 2018-09-23

**Authors:** Xuan Shi, Guannan Chen, Juan Wei, Di Feng, Yuanli Chen, Huanping Zhou, Meiyun Liu, Xin Lv

**Affiliations:** Department of Anesthesiology, Shanghai Pulmonary Hospital, Tongji University School of Medicine, Shanghai 200433, China

## Abstract

“Xuebijing Injection” (XBJ) is a traditional Chinese medicine and has been wildly used in the treatment of sepsis in China. However, few studies have reported the use of XBJ in sepsis with acute lung injury (ALI). This study aimed to investigate the therapeutic efficacy of XBJ against sepsis-induced ALI. Generally a total of 27 mice were equally randomized into three groups: a sham group was given saline before sham operation. A sepsis group received the cecal ligation and puncture (CLP) operation only. A sepsis+XBJ group, XBJ, was injected at 72, 48, and 24 h before CLP operation. The lung tissue was collected for UHPLC-Q-TOF/MS profiling analysis, biomarker identification, and pathway analysis. With the analysis of principal component analysis (PCA) and partial least squares discriminant analysis (PLS-DA), forty-five purine, amino acid, and sphingolipid metabolites in lung tissues were identified as potential biomarkers of sepsis-induced ALI, among which 22 were reversed in the sepsis+XBJ group significantly. Conclusively, our results suggest that purine metabolic pathway, glutathione metabolic pathway, sphingomyelin metabolic pathway, arachidonic acid metabolic pathway, and phospholipid metabolic pathway may be the potential therapeutic pathways to overcome sepsis-induced acute lung injury and we provided the potential mechanisms of protective effects of XBJ against ALI.

## 1. Introduction

Sepsis, characterized by high morbidity and mortality rate, has been the leading cause of critical illness worldwide [1, 2]. Among these patients, over 40% are at risk of progressing to acute lung injury (ALI), a lung disease with high morbidity [3, 4]. The treatments for sepsis-induced ALI include liquid resuscitation, antibiotic therapy, blood purification, and stem cell therapy. Despite the tremendous progress in the understanding of the mechanism of sepsis in the field of intensive medicine and other basic sciences, the mortality rate remains at a high level over the past few decades, especially in low- and middle-income countries [5]. Xuebijing injection (XBJ), a traditional Chinese herbal prescription, has been approved by the State Food and Drug Administration (SFDA) and widely used in the clinical treatment of severe sepsis. XBJ is made up of more than 20 constituents including chlorogenic acid, hydroxysafflor yellow A, rutin, and ferulic acid [6, 7]. It is an anti-inflammatory drug and has been widely used in China to block the progression of sepsis and reduce the incidence and mortality of sepsis [8]. In addition, XBJ can shorten the mean length of hospitalization, the Acute Physiology and Chronic Health Evaluation-II score (APACHEII score), White Blood Cell (WBC), C-Reactive Protein (CRP), Neutrophil (NEU), and Temperature (T^0^) of patients with sepsis. Above all, XBJ treatment can reduce the 28-day mortality of patients with severe sepsis [9]. However, the mechanisms of “Xuebijing injection” against sepsis-induced acute lung injury has is still not clear, and comprehensive mechanisms remain to be elucidated.

Metabolomics is a rapidly advancing field after the development of genomics, transcriptomics, and proteomics and can serve as a tool for the analysis of biological tissues [10]. Metabolomics can show us a better perspective to explore the entire organisms [11]. This technology can be also used to assess the holistic efficacy of traditional Chinese medicines and reveal the biomarkers of sepsis and sepsis-induced acute lung injury [12, 13]. In this study, it is the first time to use a metabonomic-based approach to explore the comprehensive changes in lung tissues in a murine model of sepsis-induced ALI and the therapeutic efficacy of XBJ against ALI.

## 2. Materials and Methods

### 2.1. Reagents

XBJ was purchased from CHASE SUN Co., Ltd (Tianjin, China). Formic acid (FA) (HPLC grade) was purchased from the Fluka Chemical Corp (Buchs, Switzerland). Methanol (HPLC grade) and acetonitrile (HPLC grade) were purchased from Merck (Darmstadt, Germany). 2-Chloro-L-phenylalanine (Sigma-Aldrich, St Louis, MO, USA) was used as an internal standard. Chromatography mass spectrometry (GC-MS) was performed using the Milli-Q system (Millipore, Bedford, MA, USA) with ultrapure water.

### 2.2. Animals and Groups

Male specific-pathogen-free C57B/L6 mice weighing 21-25g (Animal Lab Center of the Navel Medical University, Shanghai, China) were housed in a standard 12h light/dark cycle, constant temperature condition with free access to food and water. All the experiments were approved by the Animal Care and Use Committee of Tongji University (Shanghai, China). Sepsis was induced by cecal ligation and puncture (CLP) as described before [14]. Twenty-seven equally randomized into 3 groups: sham group, treated with sham operation, and saline only; model group; and treatment group, treated with 4ml/kg XBJ injection (CHASE SUN Co., Ltd. Tianjin, China) via the tail vein at 72, 48, and 24 h before CLP [15]. The lung tissues were collected from each group and stored at -80°C for future study.

### 2.3. Histology and Immunohistochemistry Analysis

After the experiments were finished, the lung tissues were perfused with normal saline (NS), inflated with 1ml formalin and removed en bloc after tracheal ligation. After that, the lung tissues were fixed in 10% PBS buffered formalin for 24 h at room temperature and embedded in paraffin. Hematoxylin and eosin (H&E) staining was performed using the standard protocol. For immunohistochemistry of ICAM and VCAM, tissue sections or cells were incubated with the primary rabbit anti-ICAM (1:2000; cell signal tech, Boston, USA) and anti-VCAM (1:2000; cell signal tech, Boston, USA) antibody, followed by the secondary goat anti-rabbit antibody (Beyotime Institute of Biotechnology, Shanghai, China). Immunohistochemical images were captured with a digital camera (Nikon, Tokyo, Japan).

### 2.4. Sample Preparation and Test

The frozen lung tissues were thawed at room temperature, added with 0.3ml ice-cold methanol containing 25*μ*g/ml 2-chloro-L-phenylalanine as the internal standard to precipitate the protein and extract the metabolites, and mixed by vortex 5 min. These samples were tranquilized for 10 min. After centrifugation at 13000rpm, 4°C for 15 min, the clear supernatant was transferred to a sampler vial.

A quality control (QC) sample was used to monitor the data acquisition performance during analysis. The QC sample was prepared by pooling aliquots from all lung samples collected in our study.

### 2.5. UHPLC-Q-TOF MS Analysis

UHPLC-Q-TOF/MS profiling analysis was performed using an Agilent 1290 Infinity LC system equipped with an Agilent 6530 Accurate-Mass Quadrupole Time-of-Flight (Q-TOF) mass spectrometer (Agilent Technologies, USA). Chromatographic separations were performed at 40°C using an Acquity UPLC HSS T3 column (2.1 mm × 100 mm, 1.8 *μ*m; Waters, Milford, MA, USA). The mobile phase was composed of 0.1% formic acid (A) and ACN modified with 0.1% formic acid (B). The total run time for one sample was 25 min including 6 min for equilibration and the optimized UHPLC elution conditions were set at: 5%B, 0-2 min; 5%-15%B, 2-10 min; 15%-30%B, 10-14 min; 30%-95%B, 14-17 min; and 95%B, 17-19 min. The injection volume was 3 *μ*L and the flow rate was set to 0.4 ml/min, and the autosampler was maintained at 4°C.

An electrospray ionization source (ESI) was operated in both positive and negative ion modes of operation. The optimized conditions used were as follows: capillary voltage, 4 kV for the positive mode and 3.5 kV for the negative mode; drying gas flow, 11 L/min; gas temperature, 350°C; fragment or voltage, 120 V; nebulizer pressure, 45 psig; and skimmer voltage, 60 V. Data were collected in the profile mode and the mass range was set from 50 to 1,100 m/z. The biomarkers were further analyzed by MS/MS, and the collision energy was set from 10 to 40 eV.

### 2.6. Statistical Analysis

The UHPLC-MS raw data in the instrument specific format were converted to common (mz.data) data format files using the Agilent Mass Hunter Qualitative software (Agilent Technologies, USA), in which the threshold was set to 0.1% and all isotope interferences were excluded. The XCMS program [16] (https://xcmsonline.scripps.edu) was used for peak extraction, retention time correction, RT alignment, and integration, in order to generate a visualization matrix. The ions were filtered to an 80% concentration [17], and to correct the MS response shift, all detected ions in each sample were normalized to total intensity. The three-dimensional (3D) data, including the sample names, RT, and m/z pairs, were imported to SIMCA-P software (version 11.0, Umetrics, Umea, Sweden) for principal component analysis (PCA) and partial least squares discriminate analysis (PLS -DA). Variable importance plot (VIP) with the threshold value of 1 was used to select metabolites. Data are represented as mean ± standard deviation (SD). The statistical significant differences were analyzed by SPSS 17.0, using Student's* t*-test and one-way ANOVA. P < 0.05 was considered statistically significant.

### 2.7. Identification of Biomarkers and Pathway Analysis

To identify the discovered biomarkers, the exact masses of ion were input into databases such as Metlin (http://metlin.scripps.edu), Human Metabolome Database (http://www.hmdb.ca/) and PubChem (http://pubchem.ncbi.nlm.nih.gov). The metabolic pathway was drawn by the Smartdraw software (SmartDraw 7.5 Hemera Technologies Inc) based on the biomarkers found in the experiment.

## 3. Results

### 3.1. Histopathology

**Figure 1:**
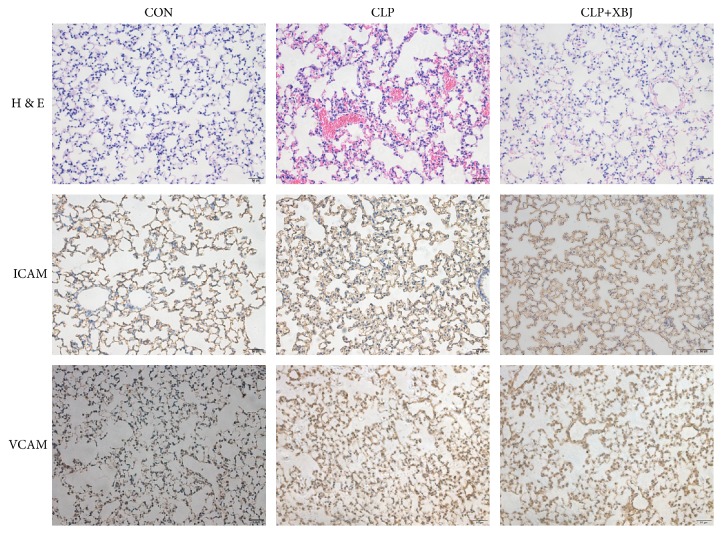
**XBJ ameliorates the acute lung injury induced by septic**. Upper panel: hematoxylin and eosin staining of lung tissues. Magnification, ×200. Middle panel: immunohistochemical staining of ICAM in lung tissues. Magnification, ×200. Lower panel: immunohistochemical staining of VCAM in lung tissues. Magnification, ×200.

To assess the protective effect of XBJ, the lung tissue sections were observed under a light microscope. As shown in Figure 1, after CLP, the lung tissues in the model group displayed thick lung septa, vascular congestion, and expansion and became congested by neutrophils. However, the severity was ameliorated after XBJ treatment as compared with the model group.

ICAM and VCAM are two types of proinflammatory adhesion molecules. As shown Figure 1, the expression of ICAM and VCAM in the treatment group was significantly decreased as compared with the model group.

### 3.2. Metabolic Profiling Analysis of Lung Tissues

**Figure 2:**
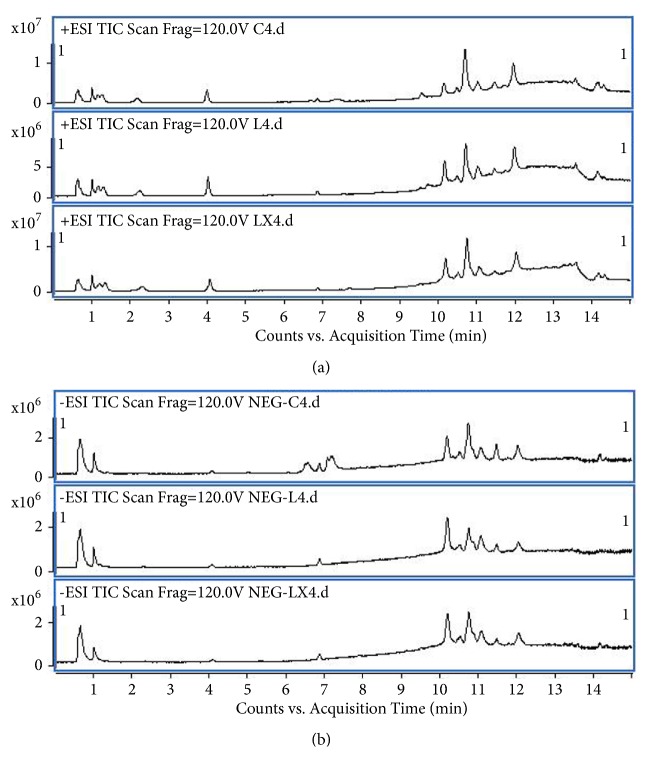
**The representative TICs in the positive and negative mode**. Representative total ion current (TIC) chromatograms of lung tissues obtained from different group of rats in the (a) positive mode and (b) negative mode. Upper panel: sham group; middle panel: model group; lower panel: treatment group.

**Figure 3:**
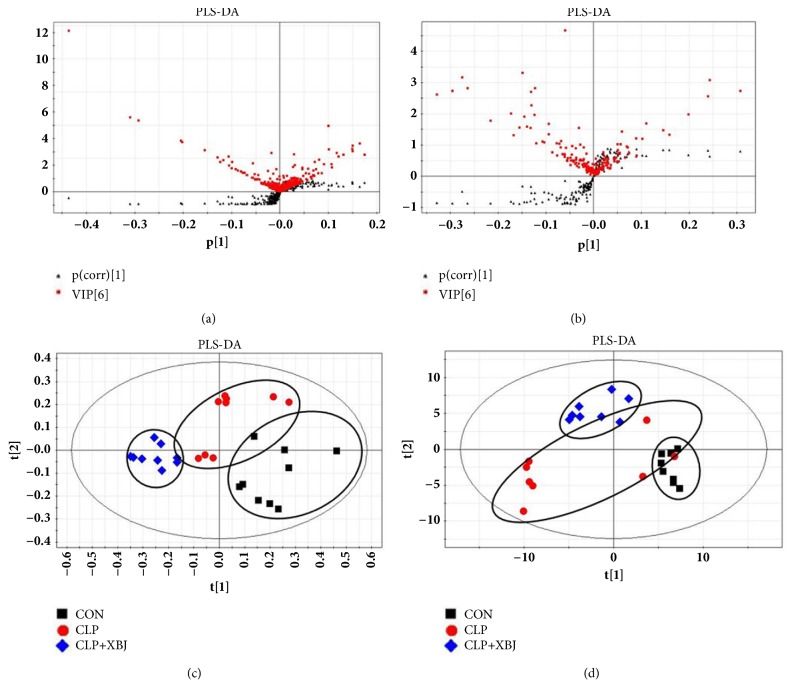
**PLS-DA and VIP-score plot in the positive and negative modes**. The combination of S- and VIP-score plot in the (a) positive and (b) negative modes showed that a greater number of values obtained from the lung tissues were further away from the origin. Score plots of the principal component analysis performed on the HPLC/MS profile of rat lung tissues obtained from the sham group (black squares), model group (red rounds), and treatment group (blue rhombus) in the (c) positive mode and (d) negative mode.

UHPLC-Q-TOF/MS data of the lung tissues were acquired based on the above methods.  Figure 2 shows the representative total ion chromatograms (TICs) in the positive mode and negative mode. These two figures displayed the general information of UHPLC-Q-TOF/MS detection. PCA score plot showed that the QC samples gathered into groups tightly, indicating that this system was stable. As shown in Figure 3, together with PLS-DA scatter plot and variable importance plots (VIPs), we defined different metabolites in the three groups. The metabolites that we chose were the points far away from the origin point in Figures 3(a) and 3(b). The metabolites with VIPs > 1.0 were considered to be important differential biomarkers. Then in Figures 3(c) and 3(d), the positive and negative modes of supervised partial least squares discriminate analysis (PLS-DA) plot were used to screen potential metabolite biomarkers from the obtained data [18]. One-way ANOVA and Tukey's post hoc test were used to assess the statistical significance.

### 3.3. Identification of Biomarkers

**Figure 4:**
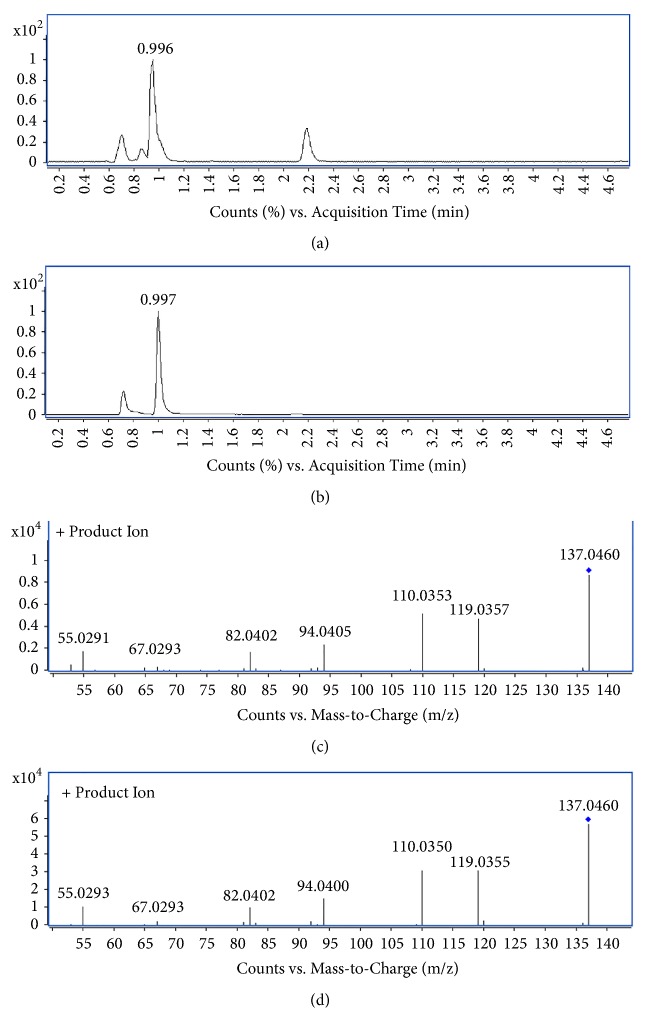
**Identification of a selected marker (m/z=137.0458)**. (a) Extracted ion chromatogram (EIC) of a commercial standard hypoxanthine (t_R_=0.996 min); (b) EIC of m/z 137.05 (t_R_=0.997min); (c) MS/MS spectrum of a commercial standard hypoxanthine; (d) MS/MS spectrum of the ion. The collision energy was 10V.

To identify biomarkers, we used the extracted ion chromatogram (EIC) to confirm the ions and then put the accurate molecular mass of ions into the Mass Bank (http://www.massbank.jp/), Metlin (http://metlin.scripps.edu/) and Human Metabolome Database (http://www.hmdb.ca/) to verify the structure that we speculated [17, 19]. Then, detected the corresponding quasi-molecular ion peak according to the retention time (RT) in the extracted ion chromatogram (EIC) of m/z 137.0458 (Figure 4(b)). Measured by Agilent MassHunter software, the accurate mass was 137.0458, and C_5_H_4_N_4_O was calculated as the most probable molecular formula. Then, the m/z of 137.0458 was identified as hypoxanthine by considering the elemental composition, fragmentation pattern, and chromatographic retention behavior, which was validated by a standard compound (Figure 4(c)). Similarly, other biomarkers have been identified according to the above method and are listed in Table 1 [20].

### 3.4. Evaluation of the Mechanism Underlying the Effect of XBJ on Sepsis-Induced ALI

**Figure 5:**
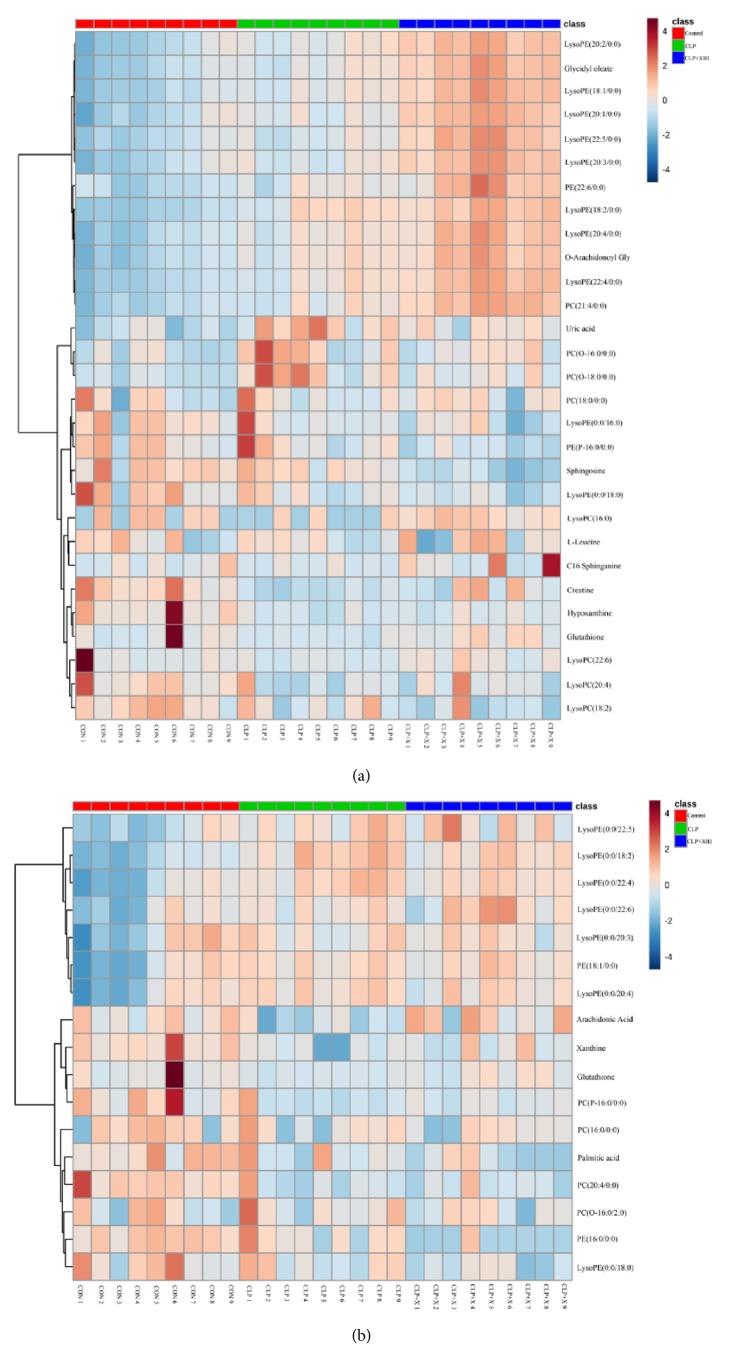
**The heatmap showed the expression levels of differential metabolites**. Heatmap visualization of the differential metabolites in these three groups in positive (a) and negative ion mode (b). In this picture, each row represents a metabolite and each column represents the expression level (red: upregulation; blue: downregulation).

**Figure 6:**
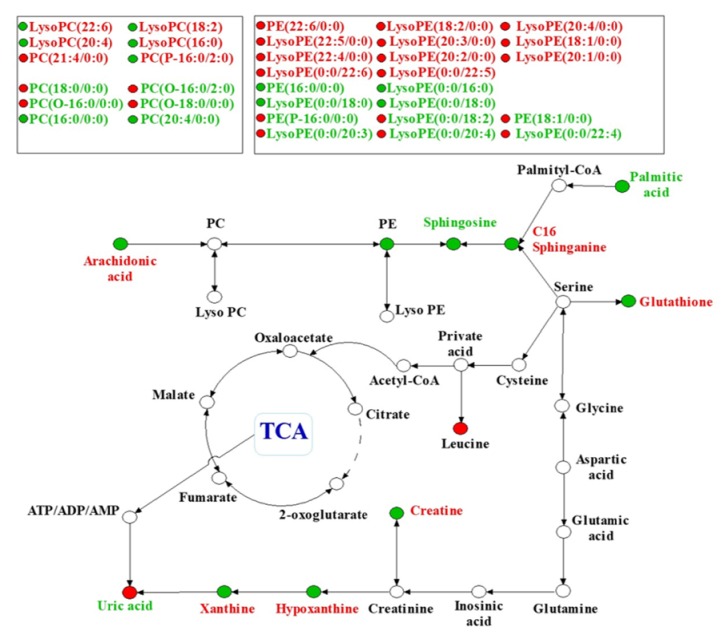
**A metabolic pathway map showed the changed metabolites and their related pathway in model group or after XBJ treatment**. The metabolic pathway networks related to the different metabolites between sepsis-induced acute lung injury and XBJ treatment groups. Red biomarkers in the networks are upregulated and the green ones are downregulated.

Finally, we identified 45 metabolites in the lung tissues to be the potential biomarkers of sepsis-induced ALI. These metabolites were valid to evaluate the protective effect of XBJ on sepsis-induced ALI. According to these statistics, we established a PCA model, R2X=0.544 and Q2=0.56, demonstrating that the model was reliable and predictive. All identified different metabolites are listed in Table 1, indicating that, among these 45 metabolites identified, 22 metabolites were reversed in the treatment group. Furthermore, the heatmap of these 45 metabolites was presented in Figure 5 both in positive and negative mode. To gain an insight into metabolic changes in different groups, we put the data into Kyoto Encyclopedia of Genes and Genomes (KEGG) pathway database (http://www.genome.jp/kegg/). The pathways of the identified metabolites are shown in Figure 6 by mainly focusing on fatty acid metabolism, amino acid metabolism, purine metabolism, and phospholipid metabolism.

## 4. Discussion

Sepsis is a common clinical condition associated with septic shock and MODS in ICUs. More than 40% patients with sepsis are at risk of progressing to ALI [4]. Previous studies showed that Gram-negative bacterial infections still play a central role in the pathogenesis of ALI and ARDS [21]. In this study, we established the sepsis-induced ALI model following the method described previously [14]. It was found in our study that the metabolites promoting oxidative stress were reduced significantly and after XBJ treatment the symptoms of ALI were alleviated, and the survival rate was improved.

No effective methods are currently available for the clinical treatment of sepsis-induced ALI. The meta-analysis performed by Shi H el al. showed that XBJ had a significant clinical effect on patients with sepsis [9]. But the extract efficacy of XBJ remains unclear. Inflammatory mediators, such as ICAM and VCAM, block the microvessels and lead to activation of leukocytes to release inflammatory mediators. These mediators can also release a large amount of oxygen free radicals [22, 23]. What is more, ICAM and VCAM are the mediators after the stimulation of TNF-*α*, IL-1, and endotoxin, leading to adhesion, aggregation, rolling, and exudation of leukocytes [24].

It was found in this study that less ICAM and VCAM gathered in the XBJ treatment as compared with the model group, indicating that XBJ was able to ameliorate sepsis-induced ALI via reducing the production of ICAM and VCAM.

Based on the results obtained from the lung tissues and comparison with the information from the databases such as HMDB, we finally identified a series of biomarkers and related pathways in the treatment group versus the model group. These pathways involved purine, glutathione, sphingomyelin, arachidonic acid metabolisms, and phospholipid metabolism.

Compared with the sham group, we observed that xanthine and hypoxanthine were downregulated, and uric acid was upregulated, and XBJ treatment could reverse these conditions.

Annette et al. [23] found that, in the process of oxidative stress, the amount of xanthine oxidase would be enhanced significantly. The xanthine oxidase (XO) could break down hypoxanthine and xanthine to uric acid and release superoxide, which is consistent with our experimental result. Previous studies [25] reported that the level of uric acid in patients with infection was elevated significantly and associated with poor prognosis. In our study, XBJ alleviated oxidative stress in lung tissues after CLP. What is more, XBJ suppressed the activation of xanthine oxidase, thus increasing the level of xanthine and hypoxanthine and decreasing the level of uric acid. These results imply that XBJ could favorably affect sepsis-induced ALI via the purine metabolic pathway.

Mervyn et al. and Novelli et al. [26–28] demonstrated that antioxidant depletion such as glutathione (GSH) was one of the most important characteristics in septic patients, which may ultimately lead to severe oxidative stress. Upregulation and activation of GSH could final ameliorate inflammation and histological injury of lung tissues [29]. In our study, we also observed the same phenomenon that the quantity of GSH was declined significantly, but the quantity of GSH was enhanced after XBJ treatment, indicating that XBJ may be a therapeutic option for sepsis-induced ALI through alleviating oxidative stress.

Sphingosine is a kind of bioactive lipid present in cell membranes. Previous studies [30] found that sphingosine exerted a critical role in signal transduction and participated in the process of growth, senescence, differentiation, and apoptosis. Published articles [26, 27, 29] showed that sphingolipid metabolism also played a critical role in oxidative stress. When sepsis occurs, it is in oxidative status, which causes severe cell membrane damage and sphingolipid loss. As a result, the body needs large amounts of sphingosine to compensate for the sphingolipid loss. It was found in our study that the amount of sphingosine in the model group was reduced significantly as compared with the control group, and XBJ treatment reversed this process, indicating that the sphingolipid metabolic pathway is activated in the condition of sepsis-induced ALI as the symbol of cell death.

Arachidonic acid is one of the important polyunsaturated fatty acids (PUFA) and can be biosynthesized from linoleic acid. Arachidonic acid plays a critical role in inflammatory metabolic pathways [31]. The data presented in a pilot study [32] showed that the activity of arachidonic acid metabolites persisted for several days in septic patients. What is more, arachidonic acid proved to activate anti-inflammatory cytokines and inhibit proinflammatory cytokines to prevent pulmonary injury [33].

Lysophosphatidylcholine (LPC) is a critical immunomodulator and has been reported to reduce mortality in septic mice [34]. Previous publication has shown that LPC concentrations on day 7 were significantly lower in nonsurvivors than in survivors and Smani et al. also reported the significant effect of LPC in severe infections [35, 36].

Meanwhile, previous studies illustrated the therapeutic effects of LPC in experimental sepsis, which included the effects on cytokine levels, on enhancing bacterial clearance, on neutrophil deactivation, and on increasing bactericidal activity of neutrophils [34, 37]. In this study, we also observed that most kind of LPC was increased in XBJ treatment group when compared with CLP group.

In summary, the disturbance of purine, glutathione, arachidonic acid, and sphingolipid metabolisms plays critical roles in the progress of sepsis-induced ALI, and XBJ treatment could reverse these metabolic disturbances in varying degrees. These results demonstrate that XBJ injection could relieve sepsis-induced acute lung injury via improving the condition of these 5 metabolic pathways.

However, Xuebijing injection consists of over 20 constituents, and some drug monomers such as ferulic acid and tanshinol can also eliminate oxygen free radicals and regulate lung inflammation [33, 38], so further studies are necessary to elucidate the detailed mechanism underlying the therapeutic efficacy of XBJ.

## 5. Conclusions

Our study demonstrated a metabonomics approach based on UHPLC/Q-TOF MS to profile the metabolic changes of sepsis-induced acute lung injury in mice and demonstrated that XBJ can alleviate sepsis-induced acute lung injury via purine, glutathione, arachidonic acid, and sphingolipid metabolisms. These pathways may be the potential therapeutic pathways to overcome sepsis-induced acute lung injury and we provided the potential mechanisms of protective effects of XBJ against ALI. However, further studies are needed for better understandings about which compounds of XBJ are involved in this pathophysiological process.

## Figures and Tables

**Table 1:** **Identification of significantly differential metabolites for lungs from control, CLP, and XBJ treated groups by UPLC-MS/TOF**. Identification of significantly differential metabolites for lung from control, CLP, and XBJ injection treated groups by UPLC-MS/TOF. Potential biomarkers related to endotoxemia induced acute lung injury and the related metabolic pathways. ^a^ Metabolites validated with standard sample. ^b^ Metabolites putatively annotated.

NO	RT/min	Detected M/Z	Theoretical M/Z	Ion	Formula	PPM	Metabolites	Fold change		Related pathway
								LPS/CON	LPS+XBJ/LPS	
1	0.70	132.0766	132.0768	[M+H]^+^	C_4_H_9_N_3_O_2_	1.51	Creatine ^a^	0.48	1.55	Glycine, serine and threonine metabolism
2	1.01	137.0458	137.0458	[M+H]^+^	C_5_H_4_N_4_O	0	Hypoxanthine ^a^	0.39	1.19	Purine metabolism
3	1.05	151.0254	151.0261	[M-H]^−^	C_5_H_4_N_4_O_2_	4.63	Xanthine ^a^	0.78	1.11	Purine metabolism
4	1.01	169.0358	169.0356	[M+H]^+^	C_5_H_4_N_4_O_3_	1.18	Uric acid ^a^	1.76	0.80	Purine metabolism
5	1.01	308.0907	308.0911	[M+H]^+^	C_10_H_17_N_3_O_6_S	1.30	Glutathione ^a^	0.32	2.57	Glutathione metabolism
6	1.01	306.0742	306.0765	[M-H]^−^	C_10_H_17_N_3_O_6_S	7.51	Glutathione ^a^	0.29	2.00	Glutathione metabolism
7	1.31	132.1018	132.1019	[M+H]^+^	C_6_H_13_NO_2_	0.76	L-Leucine ^a^	0.98	1.02	Valine, leucine and isoleucine metabolism
8	8.45	274.2743	274.2741	[M+H]^+^	C_16_H_35_NO_2_	0.73	C16 Sphinganine ^b^	0.66	2.86	Sphingolipid metabolism
9	9.80	300.2902	300.2897	[M+H]^+^	C_18_H_37_NO_2_	1.67	Sphingosine ^b^	0.93	0.56	Sphingolipid metabolism
10	10.84	339.2895	339.2894	[M+H]^+^	C_21_H_38_O_3_	0.29	Glycidyl oleate ^b^	1.39	1.43	Linoleic acid metabolism
11	14.17	255.2320	255.2330	[M-H]^−^	C_16_H_32_O_2_	3.92	Palmitic acid ^b^	0.80	0.79	Fatty acid metabolism
12	13.44	303.2319	303.2330	[M-H]^−^	C_20_H_32_O_2_	3.63	Arachidonic Acid ^a^	0.72	1.44	Arachidonic acid metabolism
13	10.11	478.2934	478.2928	[M+H]^+^	C_23_H_44_NO_7_P	1.25	LysoPE(18:2/0:0) ^b^	1.85	1.21	Phospholipid metabolism
14	10.18	526.2935	526.2928	[M+H]^+^	C_27_H_44_NO_7_P	1.33	PE(22:6/0:0) ^b^	1.28	1.34	Phospholipid metabolism
15	10.18	502.2933	502.2928	[M+H]^+^	C_25_H_44_NO_7_P	1.00	LysoPE(20:4/0:0) ^b^	1.41	1.25	Phospholipid metabolism
16	10.18	361.2741	361.2737	[M+H]^+^	C_23_H_36_O_3_	1.11	O-Arachidonoyl Glycidol ^b^	1.44	1.23	Phospholipid metabolism
17	10.21	568.3400	568.3398	[M+H]^+^	C_30_H_50_NO_7_P	0.35	LysoPC(22:6) ^b^	0.59	1.37	Phospholipid metabolism
18	10.21	544.3405	544.3398	[M+H]^+^	C_28_H_50_NO_7_P	1.29	LysoPC(20:4) ^b^	0.76	1.08	Phospholipid metabolism
19	10.24	520.3404	520.3398	[M+H]^+^	C_26_H_50_NO_7_P	1.15	LysoPC(18:2) ^b^	0.84	0.81	Phospholipid metabolism
20	10.44	528.3090	528.3085	[M+H]^+^	C_27_H_46_NO_7_P	0.95	LysoPE(22:5/0:0) ^b^	1.29	1.59	Phospholipid metabolism
21	10.57	504.3087	504.3085	[M+H]^+^	C_25_H_46_NO_7_P	0.40	LysoPE(20:3/0:0) ^b^	1.35	1.46	Phospholipid metabolism
22	10.62	496.3404	496.3398	[M+H]^+^	C_24_H_50_NO_7_P	1.21	LysoPC(16:0) ^b^	0.61	2.31	Phospholipid metabolism
23	10.69	454.2933	454.2928	[M+H]^+^	C_21_H_44_NO_7_P	1.10	LysoPE(0:0/16:0) ^b^	0.94	0.84	Phospholipid metabolism
24	10.84	480.3093	480.3085	[M+H]^+^	C_23_H_46_NO_7_P	1.67	LysoPE(18:1/0:0) ^b^	1.47	1.44	Phospholipid metabolism
25	11.01	482.3610	482.3605	[M+H]^+^	C_24_H_52_NO_6_P	1.04	PC(O-16:0/0:0) ^b^	1.63	0.79	Phospholipid metabolism
26	11.03	530.3247	530.3241	[M+H]^+^	C_27_H_48_NO_7_P	1.13	LysoPE(22:4/0:0) ^b^	1.70	1.38	Phospholipid metabolism
27	11.09	438.2985	438.2979	[M+H]^+^	C_21_H_44_NO_6_P	1.37	PE(P-16:0/0:0) ^b^	1.01	0.67	Phospholipid metabolism
28	11.16	506.3244	506.3241	[M+H]^+^	C_25_H_48_NO_7_P	0.59	LysoPE(20:2/0:0) ^b^	1.54	1.43	Phospholipid metabolism
29	11.99	524.3717	524.3711	[M+H]^+^	C_26_H_54_NO_7_P	1.14	PC(18:0/0:0) ^b^	1.06	0.97	Phospholipid metabolism
31	12.01	558.3555	558.3530	[M+H]^+^	C_29_H_52_NO_7_P	4.48	PC(21:4/0:0) ^b^	1.58	1.48	Phospholipid metabolism
32	12.01	482.3248	482.3241	[M+H]^+^	C_23_H_48_NO_7_P	1.45	LysoPE(0:0/18:0) ^b^	0.81	0.79	Phospholipid metabolism
33	12.03	508.3400	508.3398	[M+H]^+^	C_25_H_50_NO_7_P	0.39	LysoPE(20:1/0:0) ^b^	1.41	1.52	Phospholipid metabolism
34	12.35	510.3918	510.3918	[M+H]^+^	C_26_H_56_NO_6_P	0	PC(O-18:0/0:0) ^b^	2.18	0.66	Phospholipid metabolism
36	10.63	452.2777	452.2783	[M-H]^−^	C_21_H_44_NO_7_P	1.33	PE(16:0/0:0) ^b^	0.81	0.50	Phospholipid metabolism
37	10.14	476.2777	476.2783	[M-H]^−^	C_23_H_44_NO_7_P	1.26	LysoPE(0:0/18:2) ^b^	1.45	0.95	Phospholipid metabolism
38	10.87	478.2936	478.2939	[M-H]^−^	C_23_H_46_NO_7_P	0.63	PE(18:1/0:0) ^b^	1.24	0.98	Phospholipid metabolism
39	12.09	480.3088	480.3096	[M-H]^−^	C_23_H_48_NO_7_P	1.67	LysoPE(0:0/18:0) ^b^	0.91	0.83	Phospholipid metabolism
40	10.21	500.2779	500.2783	[M-H]^−^	C_25_H_44_NO_7_P	0.80	LysoPE(0:0/20:4) ^b^	1.23	0.96	Phospholipid metabolism
41	10.59	502.2930	502.2939	[M-H]^−^	C_25_H_46_NO_7_P	1.79	LysoPE(0:0/20:3) ^b^	1.15	0.99	Phospholipid metabolism
42	10.20	524.2782	524.2783	[M-H]^−^	C_27_H_44_NO_7_P	0.19	LysoPE(0:0/22:6) ^b^	1.12	1.04	Phospholipid metabolism
43	10.78	526.2934	526.2939	[M-H]^−^	C_27_H_46_NO_7_P	0.95	LysoPE(0:0/22:5) ^b^	1.33	1.02	Phospholipid metabolism
44	11.07	528.3093	528.3096	[M-H]^−^	C_27_H_48_NO_7_P	0.57	LysoPE(0:0/22:4) ^b^	1.37	0.96	Phospholipid metabolism
45	10.64	540.3303	540.3307	[M+FA-H]^−^	C_24_H_50_NO_7_P	0.74	PC(16:0/0:0) ^b^	0.87	0.91	Phospholipid metabolism
46	10.99	566.3454	566.3463	[M+FA-H]^−^	C_26_H_52_NO_7_P	1.59	PC(P-16:0/2:0) ^b^	0.66	1.06	Phospholipid metabolism
47	12.04	568.3616	568.3620	[M+FA-H]^−^	C_26_H_54_NO_7_P	0.70	PC(O-16:0/2:0) ^b^	1.05	0.92	Phospholipid metabolism
48	10.23	588.3298	588.3307	[M+FA-H]^−^	C_28_H_50_NO_7_P	1.53	PC(20:4/0:0) ^b^	0.68	0.99	Phospholipid metabolism

## Data Availability

The data used to support the findings of this study are included within the article.
